# Holistic Regulation of Angiogenesis with Chinese Herbal Medicines as a New Option for Coronary Artery Disease

**DOI:** 10.1155/2018/3725962

**Published:** 2018-08-13

**Authors:** Rong Yuan, Wei-Li Shi, Qi-Qi Xin, Ke-Ji Chen, Wei-Hong Cong

**Affiliations:** ^1^Laboratory of Cardiovascular Diseases, Xiyuan Hospital, China Academy of Chinese Medical Sciences, Beijing 100091, China; ^2^Graduate school, Beijing University of Chinese Medicine, Beijing 100029, China

## Abstract

Effectively improving myocardial blood flow and controlling atherosclerotic plaque have always been key and difficult points in the prevention and treatment of coronary artery disease (CAD). Although “therapeutic angiogenesis” is regarded as a promising approach for ischemic heart disease by improving blood flow, angiogenesis itself can induce the destabilization of atherosclerotic plaque, which reflects the double-edged role of angiogenesis. Modulating the balance of angiogenesis can be an important target for CAD treatment. Traditional Chinese medicine (TCM) emphasizes the holistic view and dynamic balance of the body. Furthermore, the principle of activating blood circulation and removing blood stasis (ABCRS) is closely connected with angiogenesis and CAD. Recent research suggests that Chinese herbal medicines for ABCRS are effective in balancing the regulation of angiogenesis. This review presents the progress of recent research on the angiogenesis regulation with Chinese herbal medicines for ABCRS in CAD. Moreover, this review demonstrates that Chinese herbal medicines for ABCRS can not only promote angiogenesis in the ischemic area to improve myocardial blood flow but also alleviate angiogenesis to stabilize plaque in atherosclerosis, which reflects the holistic regulatory role in CAD treatment.

## 1. Introduction

Coronary artery disease (CAD), also known as coronary atherosclerotic heart disease, coronary heart disease, and ischemic heart disease (IHD), is the most common cause of heart attacks [1]. According to the World Health Organization, CAD is the leading cause of death worldwide among all noncommunicable diseases [2]. Current therapeutic options are limited to pharmacological therapy, percutaneous coronary intervention, and bypass surgery. However, a large number of patients do not qualify for surgical or interventional procedures [3], and these patients mainly present with refractory angina with severe atherosclerosis in the clinic. At present, a number of studies have indicated that promoting angiogenesis is a promising approach for IHD [4], while angiogenesis in atherosclerosis induces plaque destabilization and hemorrhage [5]. Therefore, more attention should be paid to balancing the regulation of angiogenesis in myocardial ischemia and atherosclerosis.

The holistic theory of traditional Chinese medicine (TCM) aims to modulate the dynamic balance of the body. Among the different TCM therapies, activating blood circulation and removing blood stasis (ABCRS) therapy is effective in CAD treatment, with antiplatelet function, vascular endothelium protection, myocardial remodeling, and microcirculation improvement [6]. Recently, an increasing number of studies have focused on the effects of Chinese herbal medicines for ABCRS on angiogenesis in myocardial ischemia and atherosclerosis. Given the double-edged role of angiogenesis, this review aims to present the recent research progress on the regulatory role of angiogenesis by Chinese herbal medicines for ABCRS, which may provide a new angle of view with regard to the prevention and treatment of CAD.

We searched the PubMed, Chinese National Knowledge Infrastructure and Chinese Scientific Journal Database with the following keywords: “angiogenesis” OR “neovascularization” AND “coronary disease” OR “atherosclerosis” OR “myocardial ischemia/infarction” OR “Chinese medicine” OR “activating blood circulation and removing blood stasis”. We also searched related references.

## 2. The Dual Role of Angiogenesis in CAD

### 2.1. Angiogenesis

**Figure 1:**
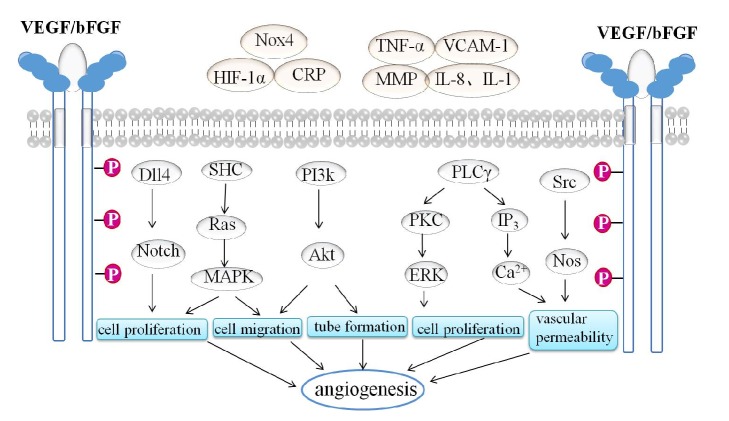
Schematic representation of major targets of angiogenesis. Nox4: nicotinamide adenine dinucleotide phosphate oxidase 4, HIF-1*α*: hypoxia-inducible factor-1*α*, CRP: C-reactive protein, TNF-*α*: tumor necrosis factor-*α*, VCAM-1: vascular cell adhesion molecule-1, MMPs: matrix metalloproteinases, Dll4: delta-like ligand 4, SHC: synthesized hydrocarbon, PKC: protein kinase C, PLC-*γ*: phospholipase C-*γ*, IP3: inositol triphosphate 3, and NOS: nitric oxide synthase.

Angiogenesis refers to the formation of new blood vessels from the preexisting vasculature [7]. Under certain conditions, various angiogenic factors are produced, and vascular endothelial growth factor (VEGF), basic fibroblast growth factor (bFGF), and their receptors are the key molecular factors. The binding of VEGF/FGF to VEGF receptor (VEGFR)/FGF receptor (FGFR) induces multiple signaling networks, such as mitogen-activated protein kinase (MAPK), phosphatidylinositol 3 kinase/protein kinase B (PI3K/Akt), extracellular regulated protein kinase (ERK), and notch pathways, and the signaling cascades result in endothelial cell (EC) survival, proliferation, migration, and tube formation [7–11]. A brief overview of the angiogenesis and the activation pathways is provided in Figure 1.

### 2.2. Angiogenesis in Myocardial Ischemia

Improving blood flow to the ischemic myocardium plays a critical role in the treatment of CAD, and angiogenesis is an important and promising means of increasing blood flow [12]. Numerous studies have shown that promoting angiogenesis therapy can improve myocardial ischemia by stimulating formation of collateral networks and increasing blood supply [4, 13]. Nox4 alleviated hypoxia/reoxygenation injury by inhibiting apoptosis and promoting angiogenesis via upregulation of HIF-1/VEGF signaling pathway [14]. Activation of the notch1 pathway also promoted coronary neoangiogenesis and revascularization, limited the extent of ischemic damage, and improved heart function [15]. Additionally, secreted VCAM-1 induced EC migration and prevented cardiomyocyte death through activation of Akt, ERK, and p38 MAPK [16]. In short, promoting angiogenesis therapy is beneficial to myocardial ischemia.

### 2.3. Angiogenesis in Atherosclerosis

Acute coronary syndrome may be related to atherosclerotic plaque rupture and thrombosis, while angiogenesis is a key factor in plaque destabilization leading to rupture [17]. Plaque neovascularization consists of a network of capillaries that arise from the adventitial vasa vasorum and extend into the intimal layer of atherosclerotic lesions, which promotes the growth of atherosclerotic lesions and plaque destabilization. Furthermore, excessive adventitial neovascularization is also one of the hallmarks of atherosclerotic plaque progression [18, 19]. Increased IL-8, IL-1, TNF-*α*, CRP, and MMP levels enhanced plaque progression and destabilization and caused intraplaque hemorrhage and rupture [20, 21]. FGFR2 overexpression in ECs resulted in increased expression of VCAM-1, which aggravated atherosclerosis [22]. The HIF pathway was associated with angiogenesis in plaque [23]. Therefore, inhibiting plaque and adventitial angiogenesis is beneficial to atherosclerosis.

### 2.4. Double-Edged Role of Angiogenesis

The “Janus phenomenon” illustrates the double-edged role of angiogenesis: when an intervention benefits proangiogenesis and collateral development, it has the potential to increase atherosclerosis, and when an intervention has antiatherosclerotic effects, it has the potential to inhibit collateral development [20, 24]. Although proangiogenesis therapy can improve blood supply in animal models, several clinical studies have not shown definite evidence of clinical efficacy of proangiogenesis in CAD, and adverse effects, including edema, inflammation, and cancer, were turned up [25, 26]. Although antiangiogenesis therapy can stabilize plaques in animal models, there has been no clinical study on antiangiogenesis therapy in atherosclerosis until now, and antiangiogenesis therapy in cancer can lead to myocardial ischemia, hypertension, and stroke [27]. Therefore, neither promotive nor inhibitory angiogenesis therapy alone is an ideal option for the treatment of CAD, and a drug that holistically regulates angiogenesis in CAD would have great potential.

## 3. The Holistic Regulatory Effects of Chinese Herbal Medicines on ABCRS

In TCM, myocardial ischemia and atherosclerotic plaque are collectively caused by blood stasis, and the ABCRS method is the main therapeutic method [6]. The TCM philosophy regards the ABCRS method as promoting blood circulation and dissipating stasis. Recent studies have also verified that Chinese herbal medicines for ABCRS have effects on improving microcirculation and hemorheology indices, increasing blood flow, regulating endothelial function, and inhibiting the proliferation of vascular smooth muscle cells [28]. A large number of studies have indicated that Chinese herbal medicines for ABCRS can regulate angiogenesis in myocardial ischemia and atherosclerosis. Therefore, the ABCRS method might have holistic regulatory effects on angiogenesis in CAD.

### 3.1. Proangiogenic Effect

Previous studies have reported that the Xiongshao capsule and Guanxin No. 2 can promote angiogenesis in the ischemic region and increase blood supply by increasing the expression levels of VEGF and bFGF [29, 30]. Tongxinluo can promote angiogenesis in the peri-infarct area and increase blood flow to the myocardium by downregulating Nox4 and by upregulating VEGF and endothelial NOS-mediated angiogenesis through the PI3K/Akt signaling pathway [31]. In addition, Qishen Yiqi dripping pills, Xuefu Zhuyu formula, Shu-mai-tang, ShenZhuGuanXin granules, flowers of Panax notoginseng, salvianolic acid B, Xuesetong soft capsules, Radix paeoniae rubra 801, Danhong injection, and Spatholobi caulis can also protect the ischemic myocardium through the activation of VEGF and the promotion of angiogenesis [32–42] (Table 1).

### 3.2. Antiangiogenesis Effect

Previous studies have determined that Tongxinluo can inhibit adventitia neovascularization and decrease microvessel density in atherosclerosis by inhibiting expression of VEGF through the p38MAPK signaling pathway [43, 44]. Simiao Yongan decoction can suppress vasa vasorum neovascularization and stabilize plaques by decreasing the expression levels of HIF-1*α*, MEK1/2, and ERK1/2 [45]. In addition, Xiongshao capsule, Huoxue capsule, Shumai capsule, modified salvia decoction, Panax notoginseng saponins, Ruanmailing, Salvianolic acid B, Guishaotongluo, and red yeast rice can also alleviate angiogenesis and attenuate atherosclerosis by decreasing VEGF expression [46–54]. Moreover, Buyang Huanwu decoction can promote microvessel maturation and decrease the incidence of plaque rupture by increasing the expression levels of bFGF and PDGF [55] (Table 2).

### 3.3. Holistic Regulatory Effects

Chinese herbal medicine for ABCRS has a holistic regulatory effect on angiogenesis. It has been reported that Rhodiola rosea and Shexiang Baoxin Pill can promote angiogenesis and increase myocardial microvessel density by increasing the expression levels of HIF-1*α*, VEGF, VEGFR2, and CD34 while inhibiting vessel growth and decreasing plaque area in atherosclerosis by reducing these indexes in the aorta [56, 57]. Another study has shown that the Xuefu Zhuyu decoction inhibits cell proliferation at certain concentrations and induces tube formation to a limited degree at low concentrations over a short time frame, suggesting that the Xuefu Zhuyu decoction controls angiogenesis in a different manner from that of the continuous function of VEGF [58]. In short, these studies imply that Chinese herbal medicines for ABCRS may have effects on balancing the regulation of angiogenesis and are thus safe for the coexistence of both myocardial ischemia and atherosclerotic lesions.

## 4. Discussion

The relationship between angiogenesis and CAD is double-sided [4]; balancing the contradictory angiogenesis effects might facilitate drug efficacy in CAD. Chinese herbal medicines have an advantage over regulating the balance of the body in different pathological states. The data reviewed here suggest that Chinese herbal medicines for ABCRS result in holistic regulatory effects and providing a new option for treating CAD. The mechanisms may be related to the multicomponent nature of Chinese herbal medicines, which may exhibit different effects in different pathological tissues through multiple targets and pathways. However, the deep mechanisms are complex and have yet to be clearly elucidated.

Modern research has indicated that angiogenesis within the vasa vasorum is characterized by a network of immature and leaky vessels, which plays an important role in plaque progression. Creating a mature network and normalizing plaque vessels may potentially minimize the risk of plaque hemorrhage [59]. In addition, EC metabolism including hypoxia-related fatty acid oxidation and glycolysis has gained attention as a therapeutic target for angiogenesis [60]. Therefore, interfering with vessel normalization/maturation and EC metabolism may be new directions to study the holistic regulatory effects of Chinese herbal medicines, which will bring new ideas to the clinical prevention and treatment of CAD.

To date, numerous studies have focused on angiogenesis in a myocardial ischemia model only or an atherosclerosis model only, while studies investigating angiogenesis in the context of both pathological changes and drug interventions are rare; there are mainly a few studies in this area [3, 56, 57, 61, 62]. Therefore, a desire exists to use compound models to study the double-edged roles of angiogenesis, therapeutic interventions, and mechanisms, especially of the holistic regulatory effects of Chinese herbal medicines. In addition, we can screen the drugs and active components that can both promote ischemic angiogenesis and inhibit proliferative angiogenesis by network pharmacology and pharmacodynamics and further identify the targets and signaling pathways of holistic regulation on angiogenesis.

Until now, clinical studies have mainly focused on promoting angiogenesis, while inhibiting angiogenesis in atherosclerosis is in the experimental stage and has not been applied in clinical practice. Hence, future work remains to be done to validate the clinical results. Meanwhile, diseases are complex in patients with medications and multiple risk factors. Therefore, clinical studies on long-term follow-up after angiogenesis-targeted therapy are worthy of investigation. In addition, although clinical trials have been conducted to evaluate the efficacy of Chinese herbal medicines in CAD, there are just a few clinical studies on the regulation of angiogenesis using Chinese herbal medicines [34, 49, 54]. Hence, more clinical trials will be required to study and to determine the best means of therapeutic angiogenesis.

In conclusion, future studies are needed to investigate the holistic regulatory effects of Chinese herbal medicines for ABCRS on angiogenesis in terms of both basic studies and clinical research, and the mechanisms of the herbs involved need to be uncovered.

## Figures and Tables

**Table 1:** Proangiogenic effects of Chinese herbal medicines for ABCRS.

**Study ID**	**Objects**	**Chinese herbs and formulae**	**Study design**	**Composition of formula **	**Therapeutic effect and mechanism**
Chen et al. [29]	8 rats	Xiongshao capsule	Gavage for 6 weeks	Ligusticum chuanxiong Hort., Radix paeoniae rubra	Increase the expression levels of VEGF and bFGF, promote angiogenesis, enhance myocardial blood supply, improve cardiac function

Zeng et al. [30]	10 rats	Guanxin No. 2	Gavage with 20 g/kg/d for 28 days	Ligusticum chuanxiong Hort., Radix paeoniae rubra, Salvia miltiorrhiza Bunge, Carthamus tinctorius Linn., Dalbergia odorifera T. Chen	Increase the expression levels of VEGF and bFGF, promote angiogenesis, compensate blood supply to the heart

Wang et al. [31]	12 mice	Tongxinluo	Gavage with 0.38 g, 1.5 g/kg/d for 7/30 days	Panax ginseng C. A. Meyer, Hirudo nipponia Whitman, Scorpio, Radix paeoniae rubra, Periostracum Cicadae, Eupolyphaga Seu Steleophaga, Scolopendra subspinipes, Santalum album Linn.	Increase the expression levels of VEGF, HIF-1*α*, eNOS, PI3K, Akt and ERK, promote angiogenesis, improve cardiac function, ameliorate cardiac remodeling

Yao [32]	ECs	Qishen Yiqi dripping pills	Drug serum for 7 days	Salvia miltiorrhiza Bunge, Astragalus membranaceus (Fisch.) Bunge, Dalbergia odorifera T. Chen, Panax notoginseng (Burk.) F. H. Chen	Increase the expression level of ERK, increase proliferation, migration and tube formation of ECs

Zhang et al. [33]	8 rats	Xuefu Zhuyu formula	Gavage with 13.68 g/kg/d for 7 days	Angelica sinensis (Oliv.) Diels, Rehmannia glutinosa Libosch, Semen Persicae, Carthamus tinctorius Linn., Radix paeoniae rubra, Bupleurum chinensis DC., Ligusticum chuanxiong Hort., Achyranthes bidentata Blume, Platycodon grandiflorus (Jacq.) A. DC.	Increase the expression level of VEGF, promote angiogenesis, protect myocardium
Li et al. [34]	40 patients		12 grains/d, po for 4 weeks		Increase levels of serum VEGF and bFGF, relieve angina and signs of blood stasis

Yin et al. [35]	24 rats	Shu-mai-tang	Gavage with 1.71 g/kg/d for 2, 4 weeks	Astragalus mongholicus Bunge, Salvia miltiorrhiza Bge, Panax notoginseng (Burk.) F.H. Chen, Hirudo nipponica Whitman, Eupolyphaga sinensis Walker, Moschus berezovskii Flerov, Trichosanthes kirilowii Maxim	Increase the expression levels of VEGF and platelet-derived growth factor, increase microvessels and arterioles in ischemic areas

Xu et al. [36]	30 rats	ShenZhuGuanXin granules	Gavage with 630, 1260, 3981.6 mg/kg/d for 4 weeks	Radix Ginseng, Radix Panacis quinquefolii, Radix Notoginseng, Hirudo, Rhizoma Pinelliae, Rhizoma Atractylodis, Folium Nelumbinis,	Increase the expression level of VEGF, increase microvessel density, attenuate infarct size, improve cardiac hemodynamic function

Yang et al. [37]	12 rats	Panax notoginseng	Gavage with 25, 50 mg/ml/d for 2 weeks	Panax Notoginseng (Burk.) F.H. Chen	Increase the expression levels of HIF-1, VEGF and VEGFR2, increase blood vessel density

He et al. [38]	15 rats	Salvianolic acid B	Gavage with 100 mg/kg/d for 4 weeks	Salvianolic acid B	Increase the expression level of VEGF, promote angiogenesis, improve myocardial microcirculation

Wang et al. [39]	8 rats	Xuesetong soft capsules	Gavage with 0.4 g/kg/d for 6 weeks	Notoginseng total saponin	Increase the expression level of VEGF, increase microvessel density

Liu et al. [40]	10 rats	Radix paeoniae rubra 801	Gavage with 16.2 mg/kg/d for 14 days	Propyl gallate	Increase the expression levels of NO, VEGF and bFGF, increase capillary density, improve myocardial ischemia

Chen et al. [41]	25 rats	Danhong injection	Intramuscular injection with 0.76 ml/kg/d for 28 days	Radix et Rhizoma Salviae Miltiorrhizae, Flos Carthami	Increase the expression level of VEGF, increase blood vessel density, decrease ratio of infarct, improve cardiac function

Zhou et al. [42]	zebrafish embryos and ECs	Spatholobi caulis	3, 10, 30 and 100 *μ*g/ml embryo medium for 24 h in zebrafish embryos; drug serum for 24 h in ECs	Caulis Spatholobi	Increase the expression levels of VEGFRs and MAPKs, increase subintestinal vessel sprouting, promote cell proliferation and migration, increase sprout intensity

**Table 2:** Antiangiogenic effect of Chinese herbal medicines for ABCRS.

**Study ID**	**Objects**	**Chinese herbs and formulae**	**Study design**	**Composition of formula **	**Therapeutic effect and mechanisms**
Ma et al. [43]	25 apoE-/- mice	Tongxinluo	Gavage with 0.38, 0.75, 1.5 g/kg/d for 5 weeks	Panax ginseng C. A. Meyer, Hirudo nipponia Whitman, Scorpio, Radix paeoniae rubra, Periostracum Cicadae, Eupolyphaga Seu Steleophaga, Scolopendra subspinipes, Santalum album Linn.	Decrease the expression level of VEGF, inhibit vasa vasorum proliferation, decrease microvessel density, reduce plaque areas

Liu et al. [44]	15 rabbits	Tongxinluo	Gavage with 0.6 g, 0.3 g/kg/d for 4 weeks	Panax ginseng C. A. Meyer, Hirudo nipponia Whitman, Scorpio, Radix paeoniae rubra, Periostracum Cicadae, Eupolyphaga Seu Steleophaga, Scolopendra subspinipes, Santalum album Linn.	Decrease the expression level of CD31, inhibit p38MAPK pathway, inhibit adventitia neovascularization

Li et al. [45]	15 apoE-/- mice	Simiao Yongan decoction	Gavage with 11.7 mg/kg/d for 8 weeks	Lonicera japonica Thunb., Scrophularia ningpoensis Hemsl., Angelica sinensis (Oliv.) Diels, Glycyrrhiza uralensis Fisch.	Decrease the expression levels of HIF-1*α*, CD34, MEK1/2 and ERK1/2, suppress vasa vasorum neovascularization, stabilize plaques

Zhang et al. [46]	10 rabbits	Xiongshao capsule	Gavage with 0.24 g, 0.48 g/kg/d for 6 weeks	Ligusticum chuanxiong Hort., Radix paeoniae rubra	Decrease the expression level of VEGF in plaque, reduce plaque areas

Ren et al. [47]	16 rabbits	Huoxue capsule	Gavage with 0.5 g, 1.5 g/kg/d for 20 weeks	Astragalus membranaceus (Fisch.) Bunge, Semen Persicae, Carthamus tinctorius Linn., Achyranthes bidentata Blume, Spina Date Seed, Ligusticum chuanxiong Hort., Radix paeoniae rubra, Citrus aurantium L.	Inhibit the expression levels of VEGF and VCAM-1 in plaque, reduce intima /tunica media thickness ratio

Qiao [48]	10 apoE-/- mice	Shumai capsule	Gavage with 700 mg, 3500 mg/kg/d for 12 weeks	Astragalus mongholicus Bunge, Salvia miltiorrhiza Bge, Panax notoginseng (Burk.) F.H. Chen, Hirudo nipponica Whitman, Eupolyphaga sinensis Walker, Moschus berezovskii Flerov, Trichosanthes kirilowii Maxim	Decrease the expression levels of VEGF, VEGFR, HIF-1*α* and Nox4, reduce plaque areas

Pan et al. [49]	30 patients	Modified salvia decoction	22 g/d po for 6 months	Salvia miltiorrhiza Bunge, Santalum album Linn., Ligusticum chuanxiong Hort., Radix paeoniae rubra, Angelica sinensis (Oliv.) Diels, Rehmannia glutinosa Libosch, Carthamus tinctorius Linn.	Decrease serum levels of VEGF, MMP-9 and CRP,reduce carotid intima media thickness, reduce plaque areas

Qiao et al. [50]	10 apoE-/- mice	Panax notoginseng saponins	Gavage with 60 mg/kg/d for 12 weeks	Panax notoginseng (Burk.) F.H. Chen	Decrease the expression levels of VEGF, CD34 and Nox4, alleviate plaque angiogenesis, reduce plaque areas

Zeng et al. [51]	10 apoE-/- mice	Ruanmailing	Gavage with 20 g/kg/d for 12 weeks	Fallopia multiflora (Thunb.) Harald., Rehmannia glutinosa (Gaetn.) Libosch. ex Fisch. et Mey, Lycium chinense Miller, Panax ginseng C. A. Meyer, Salvia miltiorrhiza Bunge, Angelica sinensis (Oliv.) Diels, Ligusticum chuanxiong Hort.	Decrease the expression levels of VEGF, bFGF and CD105, inhibit plaque angiogenesis, stabilize plaques

Zheng et al. [52]	10 apoE-/- mice	Salvianolic acid B	Gavage with 80, 160 mg /kg/d for 8 weeks	Salvianolic acid B	Decrease the expression levels of CD31, reduce neovascularization in plaque and incidence of plaque erosion, stabilize plaques

Yin et al. [53]	24 rabbits	Guishaotongluo	Gavage with 2.08, 4.16 g /kg/d for 4 weeks	Ramulus Cinnamomi, Radix Paeoniae Alba, Salvia miltiorrhiza Bunge, Curcuma longa Linn.	Decrease the expression levels of VEGF, VEGFR-2, inhibit adventitial neovascularization

Yang et al. [54]	60 patients	Red yeast rice	175 mg/d po for 6 months	Fermentum rubrum	Decrease the serum level of VEGF, reduce the density and areas of carotid plaque

Pang et al. [55]	20 rabbits	Buyang Huanwu decoction	Gavage with 20 g/d for 4 weeks	Astragalus mongholicus Bunge, Ligusticum chuanxiong Hort., Angelica sinensis (Oliv.) Diels, Radix paeoniae rubra, Semen Persicae, Carthamus tinctorius Linn., Lumbricus	Increase the expression levels of bFGF and PDGF, promote microvessel maturation, decrease the incidence of plaque rupture, stabilize plaques
